# The relationship between gut microbiota and inflammatory response, learning and memory in mice by sleep deprivation

**DOI:** 10.3389/fcimb.2023.1159771

**Published:** 2023-05-24

**Authors:** Mengjie Zhang, Mengying Zhang, Guangning Kou, Yan Li

**Affiliations:** ^1^ School of Physical Education and Sport Science, Fujian Normal University, Fuzhou, China; ^2^ Zhengzhou University, Zhengzhou, China; ^3^ Synergetic Innovation Center of Kinesis and Health, School of Physical Education (Main Campus), Zhengzhou University, Zhengzhou, China; ^4^ Centre of Sport Nutrition and Health, School of Physical Education, Zhengzhou University, Zhengzhou, China

**Keywords:** sleep deprivation, gut microbiota, inflammation, learning and memory, cognition

## Abstract

**Objective:**

Sleep deprivation has developed into a common phenomenon, which can lead to inflammatory responses and cognitive impairment, but the underlying mechanism is ambiguous. Emerging evidence shows that gut microbiota plays a crucial role in theoccurrence and development of inflammatory and psychiatric diseases, possibly through neuroinflammation and the brain-gut axis. The current study investigated the influence of sleep deprivation on gut microbiota composition, pro-inflammatory cytokines, learning and memory in mice. Further, it explored whether changes in gut microbiota increase pro-inflammatory cytokine and induce learning and memory impairment.

**Methods:**

Healthy 8-week-old male C57BL/6J mice were randomly divided into the regular control group (RC), environmental control group (EC), and sleep deprivation group (SD). The sleep deprivation model was established by the Modified Multiple Platform Method. The experimental mice were subjected to sleep deprivation for 6h/d (8:00 am∼14:00 pm) in a sleep deprivation chamber, and the duration of sleep deprivation was 8 weeks. Morris water maze test to assess learning and memory in mice. Enzyme-Linked Immunosorbent Assay determined the concentrations of inflammatory cytokines. The changes in gut microbiota in mice were analyzed by 16S rRNA sequencing.

**Results:**

We found that SD mice had elevated latency of exploration to reach the hidden platform (p>0.05) and significantly decreased traversing times, swimming distance, and swimming time in the target zone when the hidden platform was removed (p<0.05). Sleep deprivation caused dysregulated expression in serum IL-1β, IL-6, and TNF-α in mice, and the difference was significant (all p<0.001). Tannerellaceae, Rhodospirillales, Alistipes, and Parabacteroides were significantly increased in SD mice. Correlation analysis showed IL-1β was positively correlated with the abundance of Muribaculaceae (r=0.497, p<0.05) and negatively correlated with the abundance of Lachnospiraceae (r=-0.583, p<0.05). The TNF-α was positively correlated with the abundances of Erysipelotrichaceae, Burkholderiaceae, and Tannerellaceae (r=0.492, r=0.646, r=0.726, all p<0.05).

**Conclusion:**

Sleep deprivation can increase pro-inflammatory cytokine responses and learning and memory impairment in mice and may be caused by the disorder of the microbiota. These findings of this study may open avenues for potential interventions that can relieve the detrimental consequences of sleep loss.

## Introduction

Sleep deprivation has developed into a common phenomenon due to the change in work conditions and lifestyle, which disrupts millions of people’s everyday life and profoundly impacts their physical and psychological performance (Killgore, 2010; Kreutzmann et al., 2015). Adequate sleep duration is a vital foundation for maintaining health, and chronic sleep deficiency has been associated with several adverse health outcomes, including higher risks of metabolic and psychiatric diseases and inflammation (Spiegel et al., 2002; Killgore, 2010; Bao et al., 2017; Irwin, 2019; Wang et al., 2020). Sleep deprivation leads to sustained activation of the inflammatory response, and inflammation is a crucial mediator of the pathogenesis of metabolic and psychiatric diseases associated with sleep deficiency (Tobaldini et al., 2017; Shi et al., 2018). Emerging evidence suggests that sleep deprivation activates astrocytes and microglia in the brain, leading to higher levels of pro-inflammatory factors and neural injury (Xue et al., 2019). Sleep deprivation is known to cause systemic impairments in immune/inflammatory processes and cognitive deficits (Irwin, 2019), but the underlying mechanism is ambiguous.

The gut-inflammation-brain axis has been an increasingly popular research focus in recent years (Wong et al., 2016; Pearson-Leary et al., 2020). The association between sleep deprivation and oxidative stress damage in the brain, inflammatory responses, and cognitive decline is also increasingly being understood. Circadian clock dysfunction can induce the activation of macrophages, stimulating the production and release of reactive oxygen species, pro-inflammatory cytokines, and macrophage inflammatory protein, considered the critical factor in neurodegenerative diseases caused by neuroinflammation (Uddin et al., 2020). Moreover, chronic sleep deprivation significantly increases the expression of interleukin-1β (IL-1β), tumor necrosis factor-α (TNF-α), inducible nitric oxide synthase (iNOS), and nitric oxide (NO) in the brain (Liu P. et al., 2020). The levels of the inflammatory factors are positively correlated with amyloid beta (Aβ42) deposition (due to more Aβ42 being produced and/or the degradation of Aβ42 being reduced), the formation of Aβ42 deposition drives the pathogenesis of Alzheimer’s disease (AD) (D'Anna et al., 2017). Alterations of the gut microbiota have been linked to the pathophysiology of psyche developmental disorders and mental disorders, for example, autism spectrum disorder (ASD), depression, and schizophrenia, etc. (Zheng et al., 2016; Sgritta et al., 2019; F. Zhu et al., 2020), which suggests that the gut microbiota affects multiple aspects of neuroendocrinological function and brain development. The gut microbiota is involved in signal transmission between the brain, constituting the gut-brain axis (Grenham et al., 2011). Intestinal flora regulates physiological processes, interacts with the enteric and central nervous systems, and affects cognitive function development and emotion alteration (Proctor et al., 2017; Simpson et al., 2021). A severe imbalance of intestinal flora in depressed mice further leads to the significant increase of interferon-γ, TNF-α and Indoleamine 2, 3-dioxygenase 1 (IDO1) in the hippocampus of mice, aggravating depression-like behaviors (Kelly et al., 2019). A clinical study reveals that the abundance of gut microbiota with anti-inflammatory is low in elderly with cognitive impairment. In contrast, the abundance of intestinal flora with pro-inflammatory is higher (Mancuso and Santangelo, 2018). These studies suggest the possibility that the gut microbiota mediates the deleterious effects of sleep loss.

Several studies have provided preliminary evidence of sleep deprivation on diseases and the relationship between diseases and gut microbiota disorders (Li et al., 2021; Wang et al., 2021; Gu et al., 2022). However, sleep deprivation on gut microbiota and gut-brain axis has yet to be thoroughly studied. Sleep deficiency can give the body higher brittleness and susceptibility as a stressor, which may increase oxidative stress index and pro-inflammatory cytokines to a certain extent. Furthermore, oxidative stress induces brain damage, and neuroinflammation is a crucial factor leading to neurodegenerative diseases (Teleanu et al., 2022). Meanwhile, gut microbiota disorders are closely related to oxidative stress and inflammation (Shandilya et al., 2022; Zhang et al., 2022). Sleep deprivation, gut microbiota, inflammation, and cognition are closely related, and their relationship is worth further investigation, which may be a direction for the prevention or treatment of relevant diseases (Wang et al., 2021). In the present study, we intend to study the effects of sleep deprivation on gut microbiota, pro-inflammatory cytokines, and the learning and memory of mice through animal experiments. To assess whether gut microbiota alterations play a role in chronic inflammation and cognitive impairment induced by sleep deprivation, to provide the scientific basis for explaining psychological problems caused by sleep disorders.

## Materials and methods

### Animal treatment

**Figure 1:**
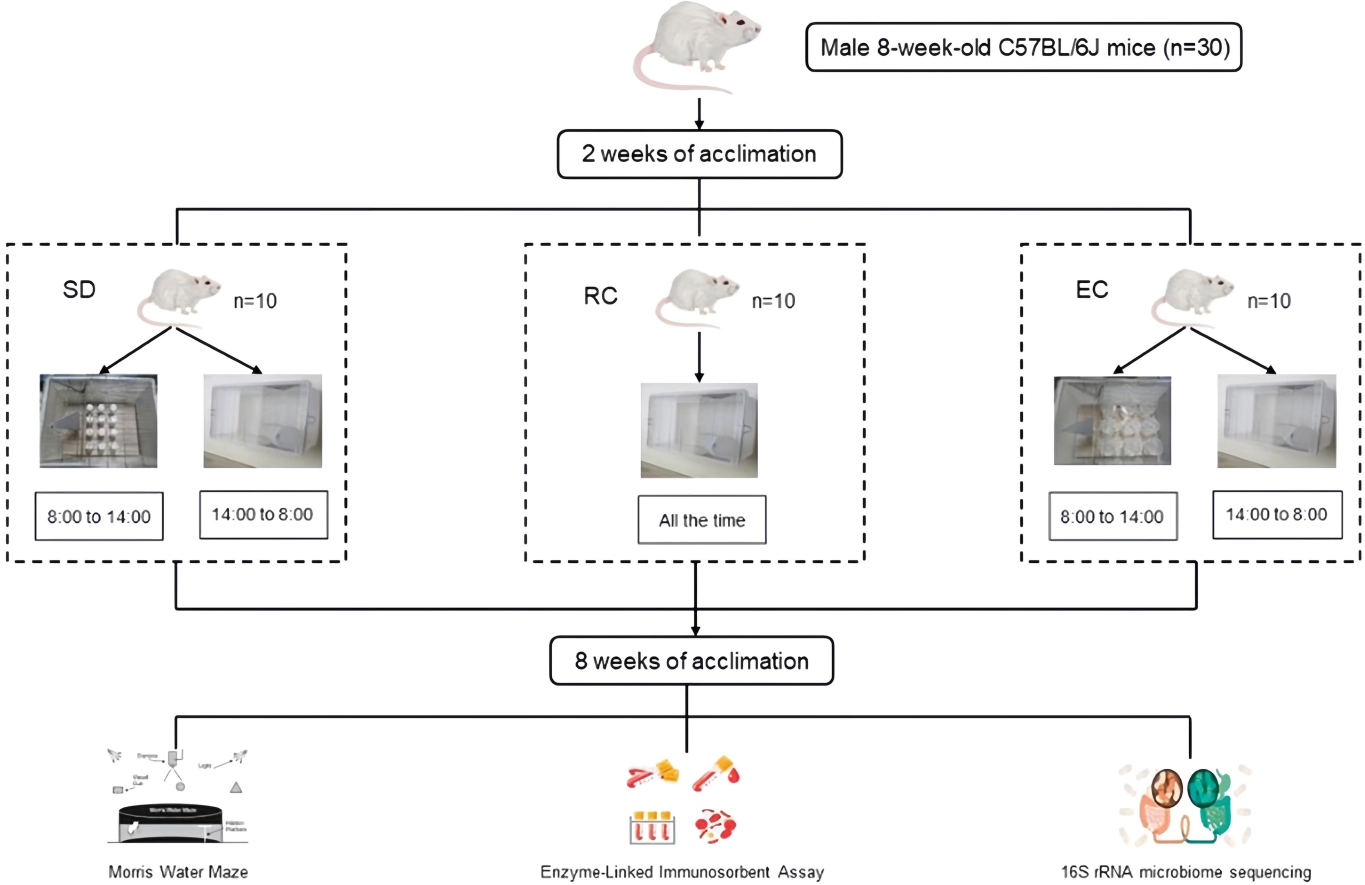
Animal treatment.

A total of 30 male 8-week-old C57BL/6J mice (No. SCXK (Yv) 2020-0005) were obtained from Henan Skbex Biotechnology Co. LTD. (Anyang, China). Mice were housed in the Medical Animal Center at the College of Public Health of Zhengzhou University under the control environment (22 ± 2°C, 45%-60% relative humidity), with a regular 12h light/12h dark cycle. The light was turned on at 8:00 and turned off at 20:00. After two weeks of acclimation, the mice were randomly divided into three groups: regular control group (RC, n=10), environmental control group (EC, n=10), and sleep deprivation group (SD, n=10). The sleep deprivation mice model was established using a Modified Multiple Platform Method (De Lorenzo et al., 2018). This method takes advantage of mice’s fear of water and places them in the sleep deprivation box. When they enter the sleep stage, their whole-body muscles will relax and wake up after falling into the water, which could realize the purpose of sleep deprivation (Machado et al., 2004). The experimental period was 8 weeks (Poroyko et al., 2016). RC mice were kept in ordinary cages during the experiment. EC mice were subjected to the environmental control box for 6 hours a day (from 8:00 to 14:00). Sleep deprivation mice were deprived of 6 hours of sleep a day in the sleep deprivation box (from 8:00 to 14:00) (Rothman et al., 2013; Saito et al., 2014). The water temperature in the box was kept at 20 to 22°C, and the water was changed once every 24 h. Food and water were given ad libitum and measured every 3 d throughout the experiment. The body weights of mice were recorded weekly. All mice had free access to food, water, and movement during this period (**Figure 1**).

### Morris water maze

**Figure 2:**
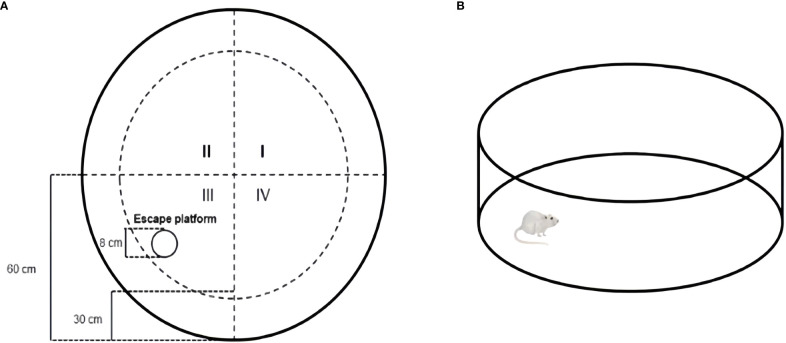
Schematic representation of Morris water maze test **(A)** Mice were subjected to the MWM test using a pool (120 cm in diameter, 30 cm in height) with opacified water and an escape platform (diameter: 8 cm); **(B)** probe test: At the last day, probe test with the same condition except for the removal of the escape platform.

The Morris water maze (MWM) was used to assess spatial learning and memory (Vorhees and Williams, 2006; Murayama et al., 2021). Mice were tested for 6 consecutive days in each trial: visible test at 1-5 days, hidden test for 6 days. The maze consisted of a round steel tank (120 cm in diameter, 30 cm in height) filled with warm water (22°C) and white nontoxic titanium dioxide added to render it opaque. The pool was divided into four quadrants (I, II, III, and IV). A white escape platform with a diameter of 8 cm was placed at a fixed location in quadrant III and submerged about 1 cm below the water surface (**Figure 2A**). The following experiments were conducted at 8:00. Experiment was always performed at the same time under the same environmental conditions. Each mouse was placed in the water in all four quadrants in a fixed order to perform four training trials per day. The interval between the two training sessions should be 15~20 min. The positioning sailing experiment should be carried out for 5 consecutive days, and latency to reach the hidden platform was measured in each mouse. The maximum trial duration was 60 s. Animals that failed to locate the hidden platform were manually guided. Once they reached the platform, they were allowed to remain there for 15 s. Memory consolidation was assessed on day 6. The platform was removed during the test, and each mouse could swim freely for 60 s (**Figure 2B**). The time of exploration, traversing times in the original platform, swimming distance, and swimming time in the target quadrant was recorded. The animal movement was tracked using the SuperMaze behavioral monitoring system (XR-Xmaze; Shanghai Xinruan Information Technology Co. Ltd.).

### Enzyme-linked immunosorbent assay

IL-1β, IL-6, and TNF-α serum concentrations were determined using an enzyme-linked immunosorbent assay (ELISA). The orbital vein blood extraction method was used to make the blood flow from the orbit into the EP tube, and the volume was 0.6-1.0 mL per sample. After 30 min at room temperature, the serum and red blood cells were rapidly separated by centrifugation at 3000 r/min for 10 min. The specific detection process follows the manufacturer’s instructions.

### 16S rRNA microbiome sequencing

Total genomic DNA was extracted from fresh fecal samples at the end of 8 weeks by using the QIAamp DNA Stool Mini Kit (Qiagen, Germantown, MD, USA) according to the manufacturer’s instructions (the fecal samples in each group were randomly selected, fecal weight >150 mg per sample, n=5 per group). The 16S rRNA of the V3-V4 area was amplified with thermal cycling, which is 2 min at 94°C, followed by 24 cycles of 30 s at 94°C, 30 s at 55°C, and 30 s at 72°C, and a final extension at 72°C for 5 min. The 16S rDNA high-throughput sequencing was carried out using the Illumina Miseq platform at TinyGene Bio-Tech Co.Ltd (Shanghai, China). Trimmed high-quality reads were clustered into Operational Units based on a >97% sequence similarity identity by UCLUST. The Silva 132 database was used as a reference for annotating taxonomic information for each sequence. Linear discriminant analysis effect size (LEfSe) was conducted to identify the biomarker species in different groups using metastats software.

### Statistical analysis

Data were analyzed using SPSS 25.0 and GraphPad Prism 8 software. Results were presented as Mean ± Standard Deviation. ANOVA followed by Tukey’s test assessed differences among more than two groups. T-test was used for the comparison of statistical significance between groups. Pearson correlation analyses analyzed the correlations of study variables. P values <0.05 were considered to be statistically significant.

## Results

### Learning and memory deficits induced by sleep deprivation

**Figure 3:**
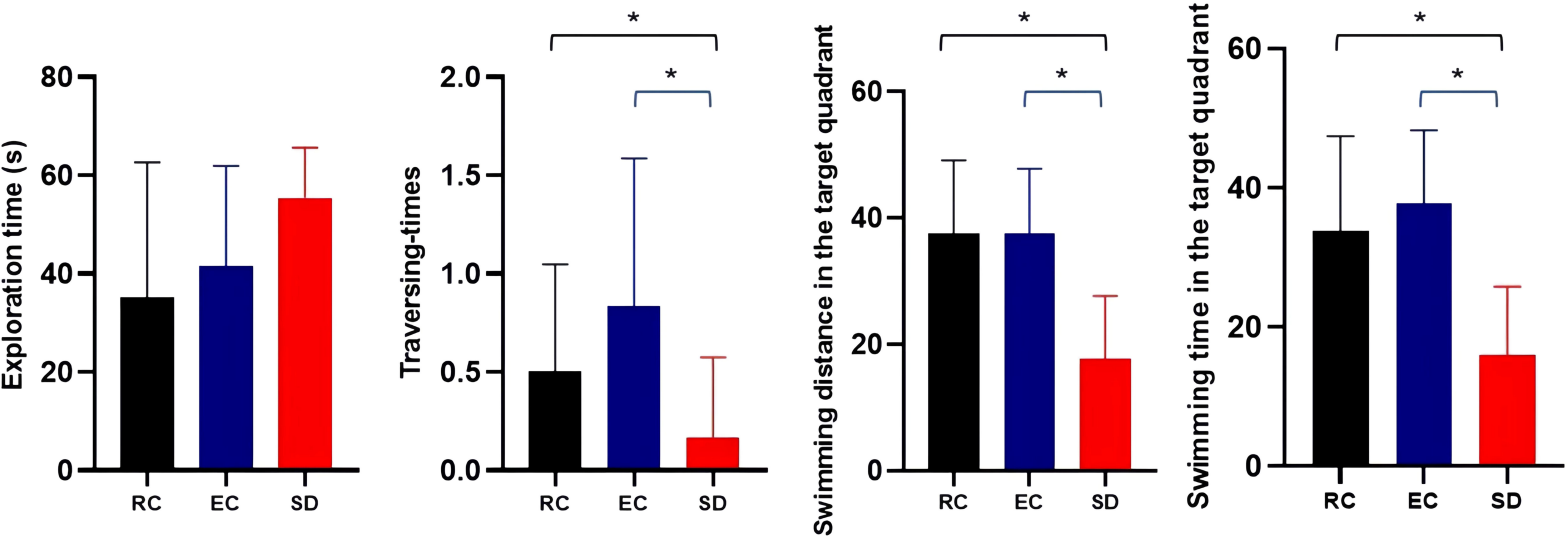
Comparison of three groups of mice in a space exploration experiment ( $\bar{x}\pm s$ ). *p<0.05.

**Figure 4:**
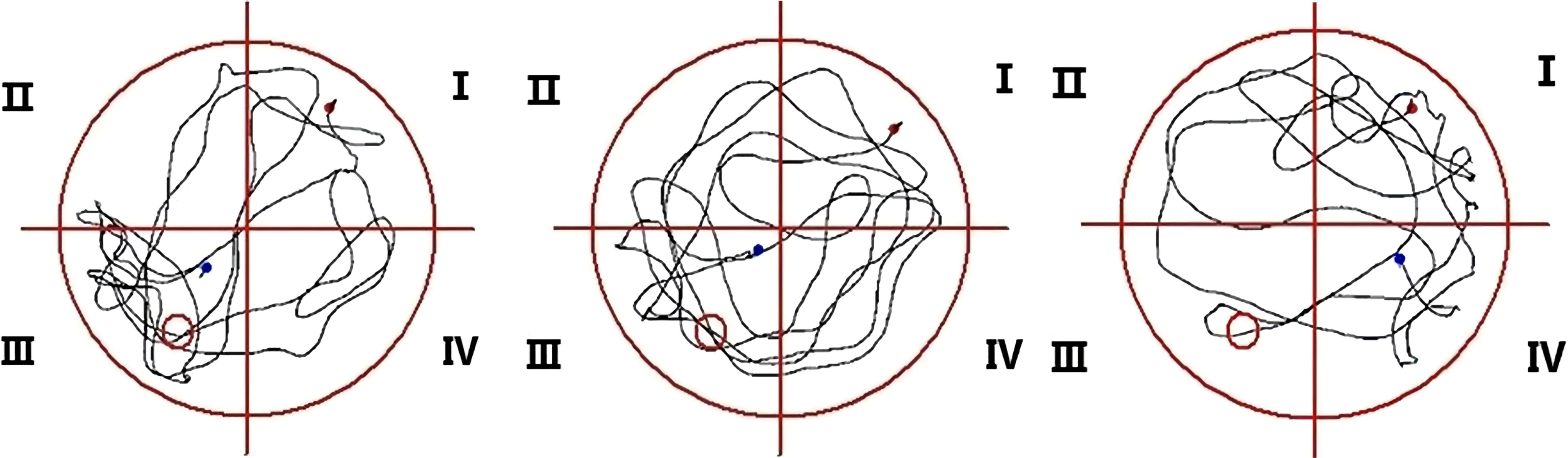
Swimming paths of three groups of mice in MWM.

The latency to reach the platform of SD mice was more prolonged than RC and EC mice during the five-day positioning navigation experiment (**Table 1**), and there were statistically significant differences on the 2nd, 3rd, 4th, and 5th days (p<0.05). In the space exploration experiment (**Figure 3**), compared with RC and EC mice, the exploration time of SD mice was extended, but there were no statistically significant differences (p>0.05). When the hidden platform was removed, there was a significantly decreased traversing times, swimming distance, and swimming time in the target zone (p<0.05). In the positioning navigation and space exploration experiments, there was no statistically significant difference between RC and EC mice (p>0.05). The swimming paths of three groups of mice in MWM showed that only SD mice had impaired spatial memory (**Figure 4**). These results suggested that sleep deprivation has impaired the cognitive function of mice.

**Table 1:** Comparison of latency in MWM positioning navigation experiment for five consecutive days.

Training days	RC (s) (n=10)	EC (s) (n=10)	SD (s) (n=10)	F	*P* values			
					p	p_1_	p_2_	p_3_
Day 1	52.26 ± 10.74	47.46 ± 8.49	53.60 ± 12.18	2.236	0.115	0.664	0.048	0.120
Day 1	42.81 ± 15.46	39.22 ± 8.94	51.00 ± 10.50	6.116	0.004	0.020	0.001	0.302
Day 1	37.19 ± 17.30	34.41 ± 5.03	44.81 ± 13.08	4.213	0.019	0.044	0.007	0.457
Day 1	25.80 ± 14.29	22.64 ± 4.59	36.13 ± 17.40	6.780	0.002	0.009	0.001	0.412
Day 1	20.30 ± 8.48	18.17 ± 2.76	35.60 ± 13.55	24.698	<0.001	<0.001	<0.001	0.435

p_1_: RC/SD; p_2_:EC/SD; p_3_:RC/EC.

### Sleep deprivation and pro-inflammatory cytokines

**Figure 5:**
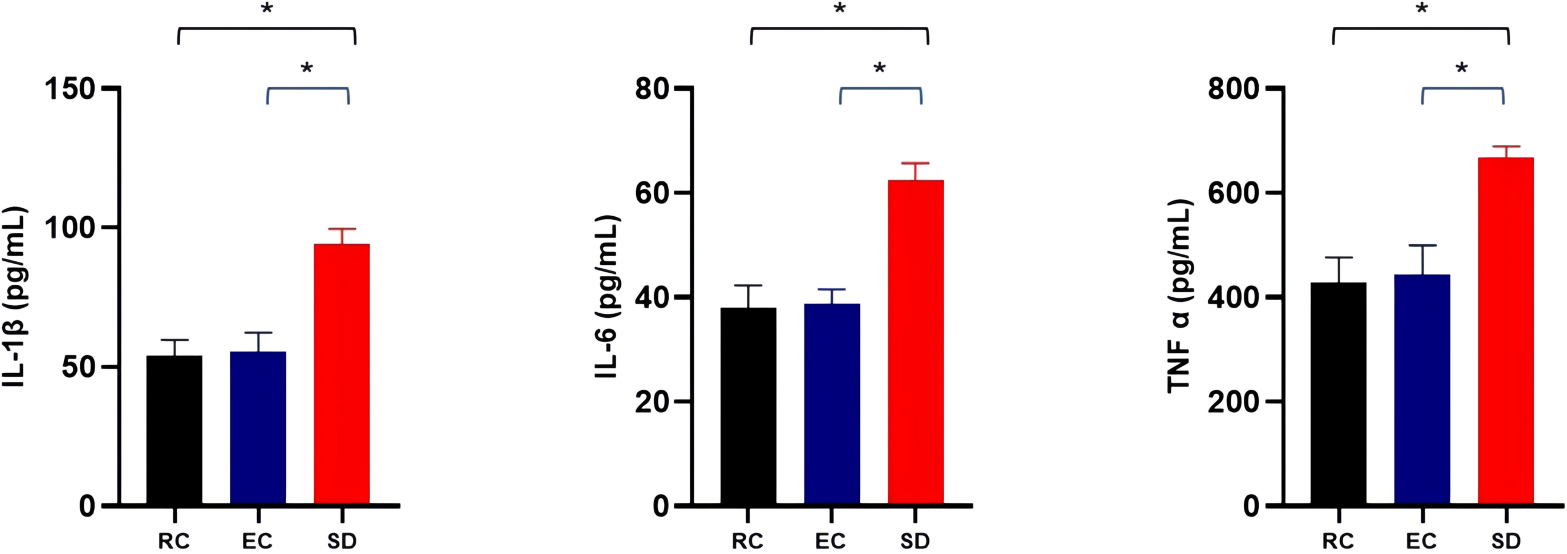
Comparison of serum levels of pro-inflammatory cytokines among three groups of mice. *p<0.05.

As shown in **Figure 5**, the detection of pro-inflammatory cytokines showed that compared with the RC and EC mice, the serum levels of IL-1β, IL-6, and TNF-α were increased by 69.28%, 61.18%, and 50.76% due to sleep deprivation, respectively, with significant significance (p<0.001). However, there were no significant differences in serum levels of pro-inflammatory cytokines (IL-1β, IL-6, and TNF-α) between RC and EC mice (p>0.05). These results suggested that sleep deprivation can increase mice’s serum levels of pro-inflammatory cytokines.

### Sleep deprivation and gut microbiota

**Figure 6:**
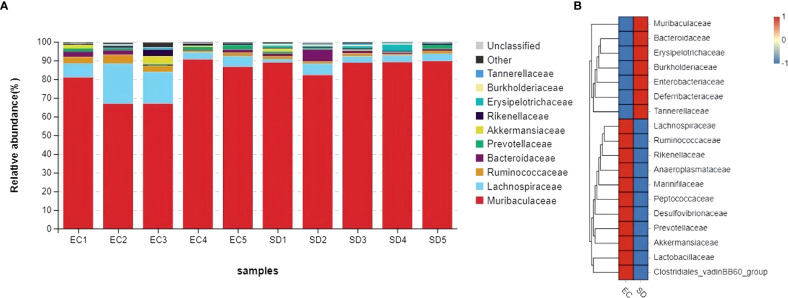
Comparative abundance of gut microbiota at family classification. **(A)**: Species distribution stack diagram **(B)**: Species abundance heat map.

**Figure 6** showed that *Muribaculaceae*, *Lachnospiraceae*, *Ruminococcaceae*, *Bacteroidaceae*, *Prevotellaceae*, *Akkermansiaceae*, *Rikenellaceae*, *Erysipelotrichaceae*, *Burkholderiaceae*, *Tannerellaceae* were top 10 in the family classification. The abundance of *Burkholderiaceae* and *Tannerellaceae* in SD mice increased by 1.8 times and 3 times, respectively, while the abundance of *Ruminococcaceae*, *Prevotellaceae*, *Rikenellaceae*, and *Akkermansiaceae* decreased by 52.9%, 41.7%, 59.3%, and 68.4%, respectively.

**Figure 7:**
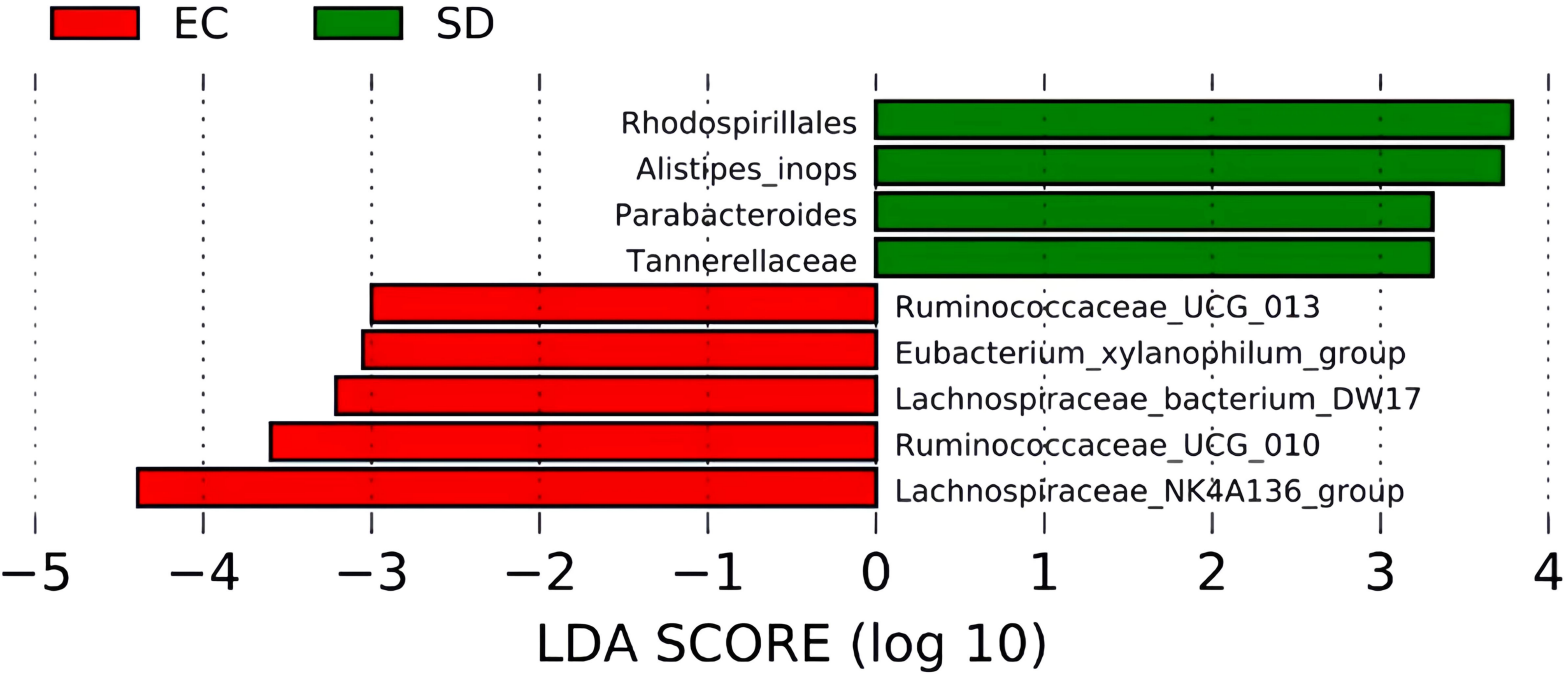
LEfSe Histogram of LDA value distribution.

As shown in **Figure 7**, 4 biomarkers were detected in SD mice. The abundance of *Tannerellaceae*, *Rhodospirillales*, *Alistipes*, and *Parabacteroides* increased significantly. However, *Ruminococcaceae_UCG_013*, *Lachnospiraceae_NK4A136_group*, *Lachnospiraceae_bacterium_DW17*, *Ruminococcaceae_UCG_010*, and *Eubacterium_xylanophilum_group* were more abundant in EC mice.

### Gut microbiota and pro-inflammatory cytokines

As shown in **Table 2**, IL-1β was positively correlated with the abundance of *Muribaculaceae* (r=0.497, p<0.05) and negatively correlated with the abundance of *Lachnospiraceae* and *Akkermansiaceae* (r=-0.583, r=-0.272, p<0.05). TNF-α was positively correlated with the abundance of *Erysipelotrichaceae*, *Burkholderiaceae*, and *Tannerellaceae* (r=0.492, r=0.646, r=0.726, all p<0.05) and negatively correlated with the abundance of *Akkermansiaceae* (r=-0.254, p<0.05).

**Table 2:** Correlation coefficient between gut microbiota and pro-inflammatory cytokines (r).

Gut microbiota	IL-1β	IL-6	TNF-α
*Muribaculaceae*	0.497^**^	0.489	0.495
*Lachnospiraceae*	-0.583^**^	-0.577	-0.556
*Ruminococcaceae*	-0.602	-0.620	-0.627
*Bacteroidaceae*	0.233	0.144	0.062
*Prevotellaceae*	-0.426	-0.496	-0.424
*Akkermansiaceae*	-0.272^**^	-0.173	-0.254^*^
*Rikenellaceae*	-0.348	-0.252	-0.232
*Erysipelotrichaceae*	0.494	0.501	0.492^*^
*Burkholderiaceae*	0.647	0.675	0.646^*^
*Tannerellaceae*	0.700	0.713	0.726^**^

*p<0.05 **p<0.01.

## Discussion

### Sleep deprivation affects the cognitive function of mice

In this study, the MWM results showed that sleep deprivation could impair the learning and memory of mice. Tuan and Lee (2019) adopted the modified multiplatform method to conduct 72-hour paradoxical sleep deprivation in mice. They found that sleep deprivation adversely affected short-term memory in a novel object recognition test, consistent with this study (Tuan and Lee, 2019). Lack of sleep will likely lead to cognitive dysfunction, especially the impact on attention (Cairney et al., 2015). Vigilant attention is a significant component of a wide range of cognitive performance, and a decrease in vigilant attention can increase the variability of the stress response. Sleep deprivation can aggravate this change (Hudson et al., 2020). AD is a devastating and irreversible cognitive impairment and the most common type of dementia. Along with progressive cognitive impairment, dysfunction of the circadian rhythms also plays a pivotal role in the progression of AD (Uddin et al., 2020). Xu also proved that sleep problems (insomnia, fragmentation, daytime dysfunction, prolonged latency, rapid eye movement sleep disorder, and excessive time in bed) were associated with a higher risk of cognitive impairment; sleep management might serve as a promising target for AD prevention (Xu et al., 2020). Disrupted sleep and circadian rhythms could increase amyloid-β (Aβ) production and decrease Aβ clearance rate (Ning and Jorfi, 2019). Moreover, acute sleep deprivation could increase tau levels in interstitial fluid. In contrast, chronic sleep deprivation could accelerate the spread of tau aggregation in neural networks (Holth et al., 2019), which leads to a decline in cognitive function and accelerates the development of AD. Several reports have also reported that decreased levels of brain-derived neurotrophic factor (BDNF) in the systemic circulation were associated with sleep disturbances (Fan et al., 2019; Furihata et al., 2020; Rahmani et al., 2020). BDNF and its receptors are widely expressed in the nervous system and play an essential role in increasing synaptic plasticity, promoting neurogenesis, and the development, differentiation, growth, and regeneration of neurons (Schinder and Poo, 2000). BDNF is involved in numerous cognitive functions, including learning and memory, and is essential for forming long-term memories (Avgan et al., 2017). These findings provide a scientific basis for new therapies to reduce the risk of cognitive dysfunction by improving sleep quality and developing ways to delay the disease in cognition impairment.

### Sleep deprivation affects the gut microbiota of mice

Although there was no significant change in microbial composition between the two groups, the relative abundance of bacteria in *Prevotellaceae*, *Rikenellaceae*, and *Akkermansiaceae* was slightly reduced compared to the control group, the relative abundance of *Enterobacteriaceae* and *Erysipelotrichaceae* was slightly increased. The human intestinal *Akkermansiaceae* family maintains intestinal integrity (Geerlings et al., 2018) and can degrade mucin. It negatively correlates with obesity, diabetes, inflammation, and metabolic disorders (Depommier et al., 2019). Gastrointestinal inflammatory diseases are frequently associated with dysbiosis, characterized by changes in gut microbial communities that include the expansion of facultative anaerobic bacteria of *Enterobacteriaceae* (*Phylum Proteobacteria*). Dysbiotic expansion of *Enterobacteriaceae* during gut inflammation could be prevented by tungstate treatment, which selectively inhibited molybdenum-cofactor-dependent microbial respiratory pathways that are operational only during episodes of inflammation (W. Zhu et al., 2018). The changes in the intestinal microbial ecosystem are related to the metabolic syndrome of most obese individuals. Studies have found that the intestinal contents of *Erysipelotrichaceae* in mice with abnormal metabolism were high, paralleled by increased expression of IL-1, IL-4, IL-6, IL-17, and IFN-γ (Pindjakova et al., 2017). Many gut microbiotas are closely related to the regulation of inflammation, and changes in the intestinal microflora caused by antibiotics could reduce the inflammatory response through metabolic status. Sun found that antibiotic exposure can significantly increase the bacterial abundance of *Prevotellaceae* and *Rikenellaceae* (Sun et al., 2019). These changes in gut microbiota can affect the inflammatory response, which is consistent with the results of this study and provides new insights into the integration of the complex network of interactions between gut microbiota and host inflammatory response.

In this study, LEfSe suggested that sleep deprivation could change the structure and composition of intestinal microflora in mice, and the abundance of microorganisms such as *Tannerellaceae*, *Rhodospirillales*, *Alistipes*, and *Parabacteroides* was significantly increased in the SD mice. However, the *Ruminococcaceae_UCG_013*, *Lachnospiraceae_NK4A136_group*, *Lachnospiraceae_bacterium_DW17*, *Ruminococcaceae_UCG_010*, and *Eubacterium_xylanophilum_group* were more enriched in EC mice. Many studies have shown that sleep deprivation can affect gut microbiota composition, but different studies suggest differences in the abundance of microbiota. Park showed that sleep deprivation could reduce *Lactobacillaceae*’s contents and increase *Erysipelotrichaceae* and *Enterobacteriaceae* contents (Park et al., 2020). Benedict demonstrated that sleep deprivation could increase the content of *Coriobacteriaceae* and *Erysipelotrichaceae* and decrease the content of *Tenericutes* (Benedict et al., 2016). The results of this study need to be more consistent with previous studies. Studies have found that *Parabacteroides_distasonis* is extremely important in SD mice, but it is one of the core bacteria in humans. Its content is negatively correlated with obesity, diabetes, and other disease states, suggesting that bacteria may positively regulate glucose and lipid metabolism (Liu S. J. et al., 2020). Further research and analysis are needed on the contradictions.

Other findings are consistent with previous research. Adriansjach found that changes in lifestyle, inflammation, and immune function could increase *Alphaproteobacteria* (Adriansjach et al., 2020). *Alistipes* is a relatively new genus of bacteria closely associated with inflammation, cancer, and mental health (Parker et al., 2020). Some studies have found that *Alistipes* has a noticeable pro-inflammatory effect, which can increase the number of Th17 cells and reduce the number of bacteria producing butyrate (Jama et al., 2019), which suggests that this species is a potential driving factor for intestinal barrier dysfunction and inflammation in patients with hypertension (Kim et al., 2018). Moreover, studies have shown that *Alistipes* can disrupt the gut-brain axis and reduce the utilization rate of serotonin. Tryptophan is a precursor of serotonin, the decrease of which is associated with depression (Kim et al., 2018). Silveira-Nunes found that serum levels of tumor necrosis factor and interleukin-6 increased in patients with hypertension, while the relative abundance of *Lactobacteriaceae* and *Ruminococcaceae* decreased significantly (Silveira-Nunes et al., 2020). *Lachnospiraceae* plays a vital role in reducing oxidative stress and preventing inflammation. Darnaud found that regenerative islet-derived protein 3α can change the colon microflora by decreasing the level of reactive oxygen species (improving the survival rate of *Lachnospiraceae*), thus improving inflammatory bowel disease (Darnaud et al., 2018). As a result, sleep deprivation can lead to an imbalance in gut microbiota, lower levels of probiotics, and high levels of harmful bacteria. This study linked sleep deprivation to the gut microbiome to identify bacterial groups sensitive to sleep disorders that could be targeted for treatment to improve sleep inadequacy for health maintenance.

### Pro-inflammatory cytokines are closely related to gut microbiota and sleep quality

The abundance of *Muribaculaceae*, *Erysipelotrichaceae*, *Burkholderiaceae*, and *Tannerellaceae* in mice was positively correlated with the levels of pro-inflammatory cytokines. The abundance of *Akkermansiaceae* and *Lachnospiraceae* was negatively correlated with the levels of pro-inflammatory cytokines. Contrary to previous studies, *Muribaculaceae* is a dominant *Bacteroidales* in mice, and it is negatively correlated with apoptosis of epithelial cells and IL-6 (Meng et al., 2019). However, this study found that *Muribaculaceae* can promote inflammation, which needs further research to explore. Although no studies have directly investigated the relationship between *Erysipelotrichaceae* and inflammation, some studies have found that the absence of congenital immune receptor NLRP12 can make mice prone to obesity, decrease insulin sensitivity and increase adipose tissue inflammation. The obesity-related *Erysipelotrichaceae* is significantly increased (Truax et al., 2018). *Burkholderiaceae* is a pathogenic bacterium. Studies have found that *Burkholderiaceae* can aggravate the inflammatory response of patients with cystic fibrosis and cause fatal lung infections (Mesureur et al., 2017). *Tannerellaceae* are oral pathogenic bacteria, and most studies focus on their relationship with oral diseases such as chronic periodontitis. The detection rate of *Tannerellaceae* at the lesion sites of periodontitis is relatively high*, so Tannerellaceae* can promote the occurrence of inflammation (Yang et al., 2007). Cao and Lang supported the results of this study, both of which found that *Akkermansiaceae* and *Lachnospiraceae* could be reduced due to inflammation (Cao et al., 2020; Lang et al., 2020).

Given that the gut microbiome interacts with pro-inflammatory cytokines, this study also sought to explore whether there is a link between inflammatory responses and gut microbes and sleep. Li found that insomnia symptoms, gut microbes, and inflammation may be related in a complex way (Li et al., 2020). Sleep is an important phenomenon related to central and peripheral immune regulation, and studies have pointed out that lack of sleep can cause mild systemic inflammation, characterized by the release of cytokines, chemokines, and acute inverse proteins, which also supports the results of this study (Hurtado-Alvarado et al., 2016). Sleep deprivation mediated inflammation may be associated with mild to moderate damage to multiple organs, adversely affecting cardiovascular and gastrointestinal health (Periasamy et al., 2015). Intestinal microbiota plays an essential role in the occurrence and development of various clinical manifestations, especially those related to the gastrointestinal tract. Evidence such as fecal transplantation to prevent inflammatory enteritis recurrence and broad-spectrum antibiotics from improving colitis supports this view (Man et al., 2011). The inflammasome regulates the composition of intestinal microbes, suggesting that the inflammatory-intestinal microbial axis may be the core of the occurrence and development of intestinal inflammation, cancer, and metabolic syndrome (Man, 2018). Sleep deprivation can cause inflammation, and pro-inflammatory cytokines can change the composition and abundance of intestinal microbes. Therefore, the intestinal microbiome can be used as an essential indicator to assist in the diagnosis of insomnia and provide a new target for treating sleep disorders.

This study found that chronic sleep deprivation had subtle effects on the gut microbiota of mice, providing a scientific basis for explaining the psychological problems caused by sleep disorders. Nevertheless, it is worth further exploring in more extensive studies, and by extension in population, to assess how sleep deprivation affects personal gut microbiota. Prospective and experimental studies are needed to confirm the possible links between sleep, gut microbiota, and cognition and to determine whether improving gut microbiota can relieve cognitive decline associated with sleep disorders. Another limitation is the current study design resulting that we could not get results that SD mice demonstrated whether resilience in their impairment of gut microbiota composition, immune function, and cognitive function after the finish of the protocol. Previous research has shown that sleep deprivation worsens inflammation and delays recovery in a mouse model of colitis (Tang et al., 2009). Moreover, sleep deprivation impairs learning-induced increases in hippocampal sharp wave ripples and associated spike dynamics during recovery sleep (Li et al., 2022). Therefore, we would analyze the gut microbiota, proinflammatory factor, and cognitive function in SD mice during sleep recovery in the subsequent experiments.

## Conclusion

In summary, sleep deprivation could cause cognitive impairment in mice and increase levels of pro-inflammatory cytokines, which may be caused by gut microbiota disorder. Sleep deprivation affected the intestinal microbiota and significantly promoted the increase of the abundance of harmful bacteria such as *Rhodospirillales* and *Alistipes*. The decrease of abundance of probiotics such as *Lachnospiraceae* and *Ruminococcaceae* in the intestine, and these microorganisms played an essential role in cognitive development. The findings of this study provide new evidence for changes in gut microbiota composition in patients with sleep disorders and provide possible directions for diagnosing and treating neuropsychiatric disorders.

## Data availability statement

The original contributions presented in the study are included in the article, further inquiries can be directed to the corresponding author. Original sequencing datasets are available in a publicly accessible repository: The original contributions presented in the study are publicly available. This data can be found here: http://www.ncbi.nlm.nih.gov/bioproject/973794 / BioProject ID: PRJNA973794.

## Ethics statement

The animal study was reviewed and approved by The Life Science Ethics Review Committee of Zhengzhou University.

## Author contributions

YL, MengjZ, and GK contributed to the study design. MengjZ and MengyZ performed the experiments. MengjZ and MengjZ analyzed the data. MengjZ wrote the manuscript. All authors contributed to the article and approved the submitted version.
